# Identification of Genetic Loci Associated with Quality Traits in Almond via Association Mapping

**DOI:** 10.1371/journal.pone.0127656

**Published:** 2015-06-25

**Authors:** Carolina Font i Forcada, Nnadozie Oraguzie, Sebastian Reyes-Chin-Wo, Maria Teresa Espiau, Rafael Socias i Company, Angel Fernández i Martí

**Affiliations:** 1 Genome Center, 451 Health Sciences Dr, University of California Davis, Davis, CA 95616, United States of America; 2 Washington State University, Irrigated Agriculture Research and Extension Center, 24106 N Bunn Road, Prosser, WA 99350, United States of America; 3 Unidad de Hortofruticultura, Centro de Investigación y Tecnología Agroalimentaria de Aragón (CITA), Av. Montañana 930, 50059, Zaragoza, Spain; Wuhan Botanical Garden of Chinese Academy of Sciences, CHINA

## Abstract

To design an appropriate association study, we need to understand population structure and the structure of linkage disequilibrium within and among populations as well as in different regions of the genome in an organism. In this study, we have used a total of 98 almond accessions, from five continents located and maintained at the Centro de Investigación y Tecnología Agroalimentaria de Aragón (CITA; Spain), and 40 microsatellite markers. Population structure analysis performed in ‘Structure’ grouped the accessions into two principal groups; the Mediterranean (Western-Europe) and the non-Mediterranean, with K = 3, being the best fit for our data. There was a strong subpopulation structure with linkage disequilibrium decaying with increasing genetic distance resulting in lower levels of linkage disequilibrium between more distant markers. A significant impact of population structure on linkage disequilibrium in the almond cultivar groups was observed. The mean *r^2^* value for all intra-chromosomal loci pairs was 0.040, whereas, the *r^2^* for the inter-chromosomal loci pairs was 0.036. For analysis of association between the markers and phenotypic traits, five models comprising both general linear models and mixed linear models were selected to test the marker trait associations. The mixed linear model (MLM) approach using co-ancestry values from population structure and kinship estimates (K model) as covariates identified a maximum of 16 significant associations for chemical traits and 12 for physical traits. This study reports for the first time the use of association mapping for determining marker-locus trait associations in a world-wide almond germplasm collection. It is likely that association mapping will have the most immediate and largest impact on the tier of crops such as almond with the greatest economic value.

## Introduction

Almond (*Prunus amygdalus* Batsch) is the most important nut crop worldwide [1]. The kernel is the edible part of the nut and is considered an important food with a high nutritional value. It may be consumed raw or cooked, blanched or un-blanched, combined and/or mixed with other nuts. Kernel quality is a priority objective in any breeding program. The physical traits and chemical components of the kernel contribute to almond quality and both must be considered in breeding programs searching for high-quality almonds [2].

The nutritional value of the almond kernel stems mainly from its lipid content, with a higher level of monounsaturated and polyunsaturated fatty acids (PUFA) than saturated fatty acids. This fat profile is highly recommended in human nutrition. Among the fatty acids, oleic and linoleic acids represent over 90% of the total lipid content [3]. The predominance of unsaturated fatty acids in almond is very important from nutritional and health viewpoints, since these fatty acids do not contribute to the synthesis of cholesterol in the human body [4]. Therefore, almond, along with other nuts, plays an important role in the human diet [2]. Almond oil contains tocopherols, an indispensable nutrient for humans and animals due to their antioxidant and radical scavenging abilities. Moreover, tocopherols are also important for the oxidative stability of vegetable oils. Hence, high tocopherol contents or an altered tocopherol composition in almond kernel oil may be a target in almond breeding for increased quality.

Although quality is often related to the chemical composition of any fruit or nut, some physical parameters must also be taken into account when evaluating quality. The physical traits of the almond nut do not affect the organoleptic characteristics of the kernel, but have a special importance in the industry because of the different steps involved in almond processing [2]. For example, shell hardness plays an important role during harvest and the industrial processes, since the shell is sometimes not well sealed through the suture line in soft-shell cultivars, leaving an entry point for dust, insects and micotoxin-producing fungi, such as *Aspergillus* producing aflatoxins [5]. The size and the shape of the almond nut must also be taken into account for designing and adjusting appropriate technologies for harvest, dehulling, transporting, classifying, processing and storing the crop. Besides, the size and shape of the kernel may define its utilization in specific commodities, such as chocolate bars, sugared almonds and sliced kernels.

It has been suggested, that most quality traits in almond are quantitatively inherited and therefore under polygenic control [6]. The heritability and genetic control of quality traits in almond been established in our germplasm [1, 7]. For example, Font i Forcada et al. [7] reported a heritability (h^2^) of 0.60 for γ-tocopherol content. Heritability estimates depend on the variation between genetic and the environmental effects [8]. For instance, a trait with a high heritability estimate indicates additive gene action [9] and little environmental influence and high genetic advance. In addition, several QTLs affecting both physical and chemical traits of almond nut have been identified [3, 10]. These results suggest a significant and positive effect of the pollen on the transmission of most chemical components of the almond kernel and also provide an understanding of genetic control to facilitate development of new cultivars for the benefit of producers, shippers and consumers in the almond industry.

Almond is the most diverse of all species in the genus *Prunus* [11]. The resulting high variability in almond [12] may not only be due to its self-incompatibility system and the use of open-pollinated seedlings in traditional almond culture, but also because almond is the most southern *Prunus* species, and thus subject to more diverse growing conditions than other stone fruits, resulting in an adaptation to more diverse microclimates. The CITA almond germplasm collection based on accessions from all over the world is a fair representation of the genetic diversity available in almond [13]. Taking this diversity into account, this collection is being used as the almond reference collection for the Spanish Plant Genetic Resources Network, the Spanish and the European Community Plant Variety Offices, and the ‘Group de Recherches et d'Études Médirerranéen pour l'Amandier’ (GREMPA).

This collection has been widely studied since its original establishment by Dr. A.J. Felipe in the 1960s. A large amount of published and unpublished data from the collection is available, such as complete descriptors of morphological traits, including tree, shoot, leaf, nut and kernel traits, bloom time, chilling and heat requirements [14], *S*_*f*_ allele diversity [15], genetic diversity [12], and kernel composition [15]. This collection was also used to start the CITA almond breeding program, where not only very important agronomical traits, such as late bloom and self-incompatibility, were assessed, but also all aspects of almond quality [1].

Association mapping (AM), also known as linkage disequilibrium (LD) mapping may complement classical linkage mapping for elucidating the genetic basis of complex traits [16]. This approach relies on the strength of association between genetic markers and phenotype. Thus, it detects and locates genes relative to an existing map of genetic markers [17]. While linkage analysis searches for associations within populations developed from bi-parental crosses, AM utilizes unstructured or loosely structured populations (e.g., a collection of varieties or landraces), case-control designs and transmission disequilibrium tests [18]. Consequently, this method detects relationships between phenotypic variation and gene polymorphism in existing germplasm and in unrelated individuals. The effectiveness of this approach is greater when information on population structure and LD is available [19]. Since many important crops have complex population structures that arose from a long domestication and breeding history [20], understanding these structures is important to avoid identifying spurious associations in association mapping. In addition, AM complements and enhances previous QTL (Quantitative trait loci) information by incorporating the effects of recombination occurring in many past generations into a single analysis [21]. Association mapping has been successfully used to identify genes involved in several traits in different plant species (maize, sunflower, lettuce, potato, and wheat), but only a few studies have been carried out in fruit tree crops, such as peach [19, 22], sweet cherry [23] and apple [24]. Consequently, AM in tree species requires both genotyping and phenotyping of large populations with unique architectures.

The purpose of our study was to phenotype the worldwide collection of almonds available at CITA with the goal of carrying out an association study to link SSR genotypes to the chemical and physical nut and kernel traits. We believe this will be one avenue to provide molecular information for exploring QTLs of important chemical and physical traits in almond. To our knowledge, this is the first report of an association study in almond.

## Results

### Phenotypic diversity

The variation observed in the chemical and physical traits was high, thus confirming the diversity in the germplasm and the representativeness of the gene pool (Table 1). Most traits displayed a normal distribution. The protein content ranged between 10 and 30% (± 4.58) of the kernel dry weight (DM) while the oil content ranged between 51 and 67% (x 3.18) of the kernel DM. The range for the main fatty acids in comparison to the total oil content was 63–80% (±3.66) for oleic acid and 11–27% (x3.20) for linoleic acid. The ranges tocopherol homologues were 331–585 mg kg^–1^ (±5.38) for α-tocopherol, 0.11–3.14 mg kg^–1^ (± 0.06) oil for δ-tocopherol, and 5–57 mg kg^–1^ (±2.69) for γ-tocopherol (Table 1). Physical traits also showed a wide range in variability (Table 1), including nut weight; 1.6 to 9.9 g (±1.43), kernel weight; 0.6 to 3.5 g (± 0.46), nut thickness; 8.9 to 19.9 mm (±1.62) and kernel thickness; 6 to 12 mm (±0.85). These results highlight the importance of the genetic background of each accession in the phenotypic profile of the nuts and kernels.

**Table 1:** Units, minimum, maximum, mean and standard deviation values for the chemical and physical traits evaluated in the almond germplasm.

Trait	Units	Minimum	Maximum	Mean	Standard deviation
α-Tocopherol	mg kg^-1^ oil	331	585	457	5.4
δ-Tocopherol	mg kg^-1^ oil	0.1	3.1	0.9	0.1
γ-Tocopherol	mg kg^-1^ oil	5	57	17	2.7
Oleic acid	% of total oil content	63	80	72	3.7
Linoleic acid	% of total oil content	11	27	19	3.2
Stearic acid	% of total oil content	1.5	3.5	2.1	0.2
Palmitic acid	% of total oil content	5.1	7.2	6.1	0.5
Palmitoleic acid	% of total oil content	0.3	0.6	0.4	0.1
Oil content	% of kernel dry weight	51	67	60	3.2
Protein content	% of kernel dry weight	10	30	20	4.6
Nut width	mm	16	29	23	2.4
Nut thickness	mm	8.9	20	16.2	1.6
Nut length	mm	25	47	33	4.3
Nut weight	gr	1.6	9.9	4	1.4
Nut T/L ratio	—	1.6	3.0	2.1	0.7
Kernel doubles	%	1	50	10	2.8
Kernel width	mm	11	19	14	1.5
Kernel thickness	mm	6	12	8	0.9
Kernel length	mm	19	30	24	2.5
Kernel weight	g	0.6	3.5	1.5	0.5
Kernel T/L ratio	—	2	4.5	3.3	0.5

### Allelic variation at SSR loci

All 40 loci analyzed showed polymorphism, producing well-defined and reproducible bands in all accessions. A total of 557 alleles were amplified from all loci scored in all 98 accessions, with an average of 13.9 alleles per locus, and a range from 2 alleles in EPDCU5100 and CPSCT018 to 23 alleles in BPPCT053 (Table 2). All primers produced a maximum of two bands per genotype in accordance with the diploid nature of almond. Genotypes showing a single band were considered homozygous at that locus. The observed heterozygosity ranged between 0.24 (BPPCT030) and 0.94 (CPPCT040), with an average of 0.66 across all 40 SSRs. Expected and observed heterozygosity values were compared with the fixation index (F) which was on the average, 0.11, ranging between -0.14 (EPDCU5100) and 0.52 (BPPCT010). The high F values observed corresponding to high homozygosity (particularly in individuals with only one band) suggests the presence of null alleles [25]. The F value was positive in 33 and negative in 7 SSR loci.

**Table 2:** Genetic parameters of 98 almond cultivars based on 40 SSR loci.

SSR	*A*	*Ae*	*Ho*	*He*	*Fis*	*I*	*PD*
BPPCT011	19	7.7	0.78	0.87	0.10	23.13	0.87
CPPCT053	23	12.5	0.83	0.92	0.09	27.12	0.92
EPDCU5100	2	1.3	0.25	0.22	-0.14	0.37	0.18
BPPCT001	15	4.5	0.39	0.78	0.50	19.95	0.79
BPPCT030	4	1.3	0.24	0.22	-0.12	0.44	0.20
CPPCT044	18	4.8	0.70	0.79	0.11	20.79	0.69
CPSCT021	14	4.8	0.69	0.79	0.13	19.45	0.92
PceGA34	13	2.4	0.56	0.58	0.03	13.77	0.61
BPPCT007	16	8.3	0.83	0.88	0.05	23.27	0.88
BPPCT039	15	11.1	0.67	0.91	0.24	24.58	0.90
CPDCT025	21	11.1	0.81	0.91	0.11	26.47	0.93
EPDCU0532	9	4.8	0.78	0.79	0.01	17.14	0.79
UDP96-008	5	2.2	0.53	0.55	0.03	0.97	0.43
BPPCT010	16	7.1	0.41	0.86	0.52	22.42	0.93
CPDCT045	17	10.0	0.90	0.90	-0.02	24.89	0.92
CPPCT005	20	10.0	0.87	0.90	0.03	25.27	0.95
EPPCU6216	16	5.9	0.75	0.83	0.09	21.37	0.84
EPPCU9168	12	4.3	0.75	0.77	0.02	19.07	0.77
PMS40	18	5.3	0.75	0.81	0.07	20.72	0.81
PS12e2	16	8.3	0.85	0.88	0.03	23.40	0.89
UDP96-003	21	10.0	0.76	0.90	0.14	24.93	0.93
UDP97-401	17	9.1	0.67	0.89	0.24	24.04	0.90
BPPCT038	11	5.6	0.78	0.82	0.03	19.25	0.81
CPPCT009	9	3.2	0.57	0.69	0.16	16.01	0.68
CPPCT040	20	12.5	0.94	0.92	-0.03	26.69	0.92
CPSCT006	7	2.6	0.64	0.62	-0.03	11.82	0.62
CPSCT022	8	1.9	0.42	0.48	0.13	0.96	0.47
PceGA25	18	6.2	0.75	0.84	0.11	21.73	0.84
BPPCT025	16	8.3	0.78	0.88	0.11	23.07	0.90
CPPCT008	7	4.0	0.71	0.75	0.04	15.12	0.72
CPPCT021	17	3.1	0.42	0.68	0.38	16.61	0.68
CPPCT047	19	6.7	0.71	0.85	0.17	23.34	0.90
CPSCT012	16	10.0	0.72	0.90	0.20	24.60	0.90
MA040	10	4.8	0.68	0.79	0.13	18.18	0.79
EPDCU3392	10	5.6	0.48	0.82	0.42	19.33	0.82
CPPCT022	19	5.3	0.59	0.81	0.27	21.23	0.81
EPPCU7340	16	8.3	0.67	0.88	0.24	22.82	0.88
PMS02	4	1.6	0.32	0.39	0.18	0.79	0.35
CPPCT006	21	11.1	0.92	0.91	-0.02	26.35	0.96
CPSCT018	2	1.7	0.55	0.41	-0.38	0.59	0.40
Mean	13.9	4.2	0.66	0.76	0.11	18.20	0.76

*A* observed number of alleles per locus, *A*_*e*_ effective number of alleles per locus, *H*_*o*_ observed heterozygosity, *H*_*e*_ expected heterozygosity, *F*_*is*_ Wright’s fixation index, *I* Shannon’s information index, *PD* power of discrimination

### Cluster analysis and grouping of accessions

**Fig 1:**
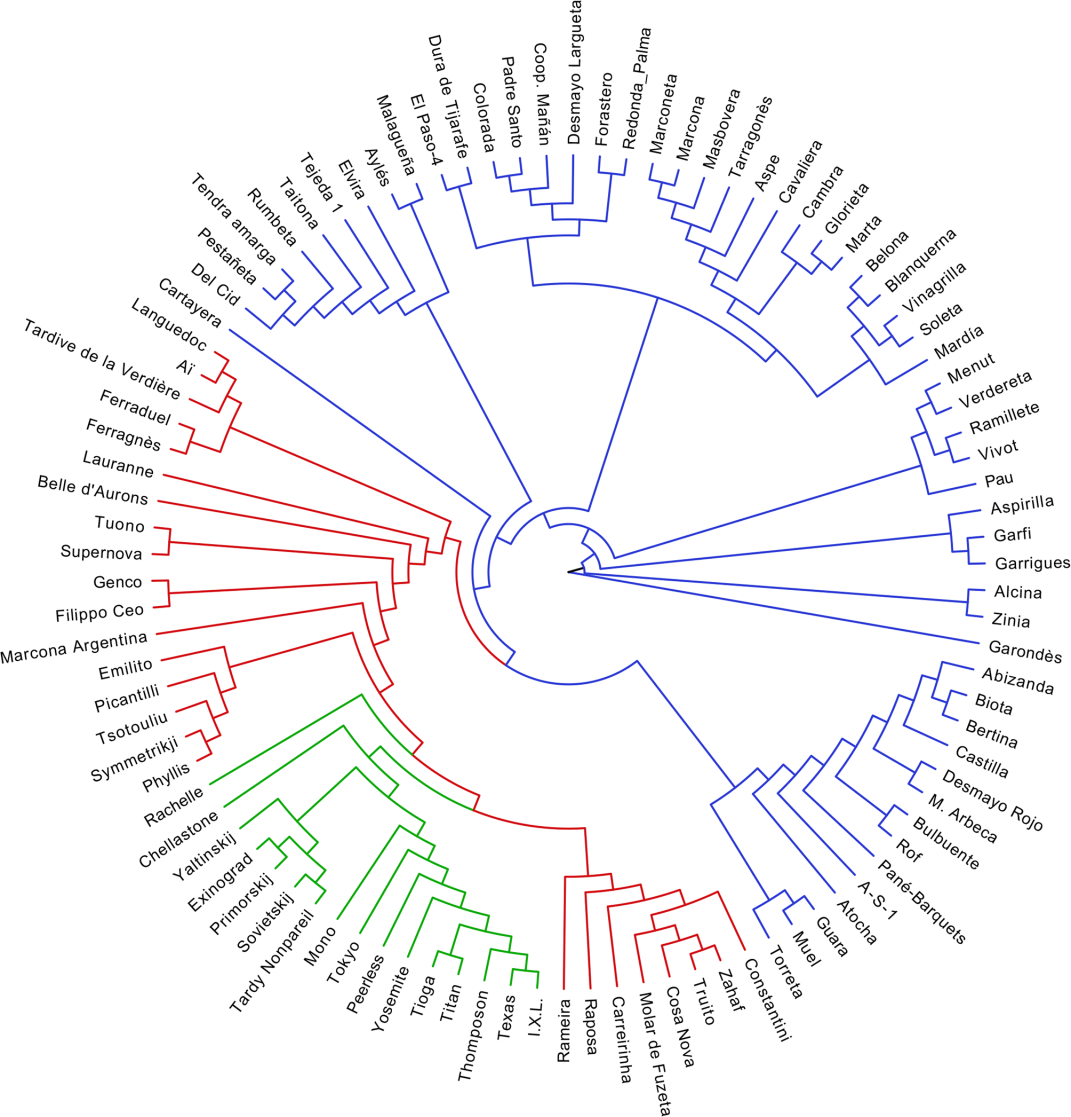
A phenogram based on Neighbor-joining analysis showing the genetic relationships of 98 CITA almond collection using 40 SSR markers. Local accessions from Spain are shown in a blue branch line; cultivars from other Mediterranean countries are shown in a red branch line while cultivars from other regions are shown in a green branch line.

Cluster analysis based on the Neighbor-joining method detected three major clusters and sub-clusters (Fig 1). In some clusters, the groupings concurred with the geographical origin of the accessions. The first and largest cluster (blue) contained only the Spanish accessions (57). This group comprises cultivars from the north-eastern part of Spain including Guara, Bertina, Muel, Desmayo Largueta or Castilla as well as cultivars from the south and south-eastern parts such as Marcona, Ramillete, Coop. Mañán, Atocha, Garrigues, Del Cid or Malagueña. The new releases from different Spanish breeding programs such as Mardía, Soleta, Masbovera, Tarragonès, Marta and Belona were also grouped in this cluster. In addition, this cluster contains a group of cultivars from the Canary Islands (El Paso-4, Colorada, Dura de Tijarafe, Padre Santo and Redonda de Palma) and the Majorca Island (Vivot, Garondès and Vinagrilla).

The second group (in red) contains 25 accessions including a pool of Mediterranean cultivars such as Constantini and Zahaf from North Africa, seven French cultivars (Belle d’Aurons, Lauranne, Ferragnès, Ferraduel, Tardive de la Verdière, Aï and Languedoc), four Italian genotypes (Tuono, Supernova, Genco and Filippo Ceo), five Greek cultivars (Picantilli, Tsotouliu, Truito, Symetriky and Phyllis), and five Portuguese cultivars (Cosa Nova, Molar de Fuzeta, Carreirinha, Raposa and Rameira). The third group (green) encompasses 16 cultivars of diverse origins, including Bulgaria (Eixinogrand) and Ukraine (Yaltinskij, Primorskij and Sovietskij), Australia (Chellastone and Rachelle), and USA (I.X.L., Texas, Thompson, Titan, Tioga, Yosemite, Peerless, Tokyo, Mono and Tardy Nonpareil).

### Population structure and linkage disequilibrium

**Fig 2:**
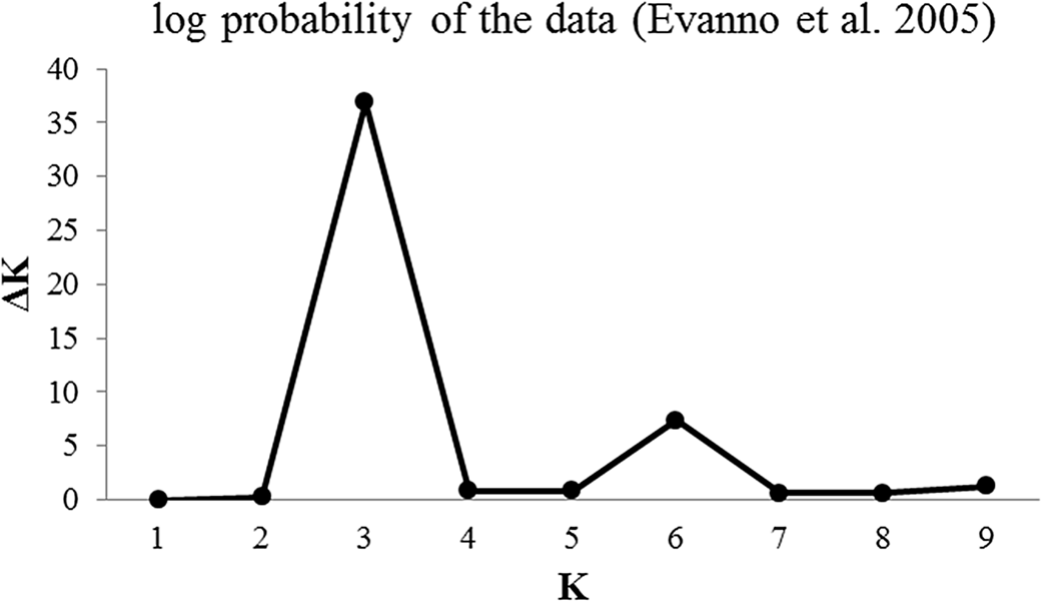
The log likelihood for each K, Ln P (D) = L (K) probability [21].

**Fig 3:**
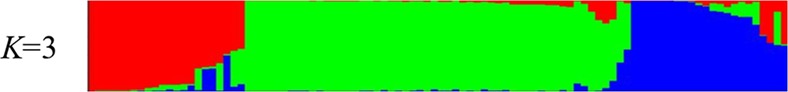
Grouping of 98 almond accessions genotyped at 40 SSR loci based on STRUCTURE analysis. Green and blue represent individuals within the subpopulations. Any blue or green bar that is not completely filled indicates admixture.

Population structure in the accessions was assessed using the STRUCTURE software package. Bar plots were obtained with different values of *K*, from two to ten (Fig 2). We also performed twenty independent runs per K value with both the MCMC replications and analysis of the rate of change in the log probability of the data (∆*K*) which displayed a maximum value at *K* = 3 [26]. The level of partitioning corresponds to a very strong differentiation into two major groups (Fig 3). The first group contained accessions from Mediterranean countries (Western-Europe), mostly from Spain, but also from Italy, France and Portugal, etc., while the second group contained accessions from non-Mediterranean countries (including America, Australia and Eastern-Europe). The proportion of genotypes assigned to each population was not symmetric, and many accessions were assigned to one population or the other, indicating that population structure exists [27].

**Fig 4:**
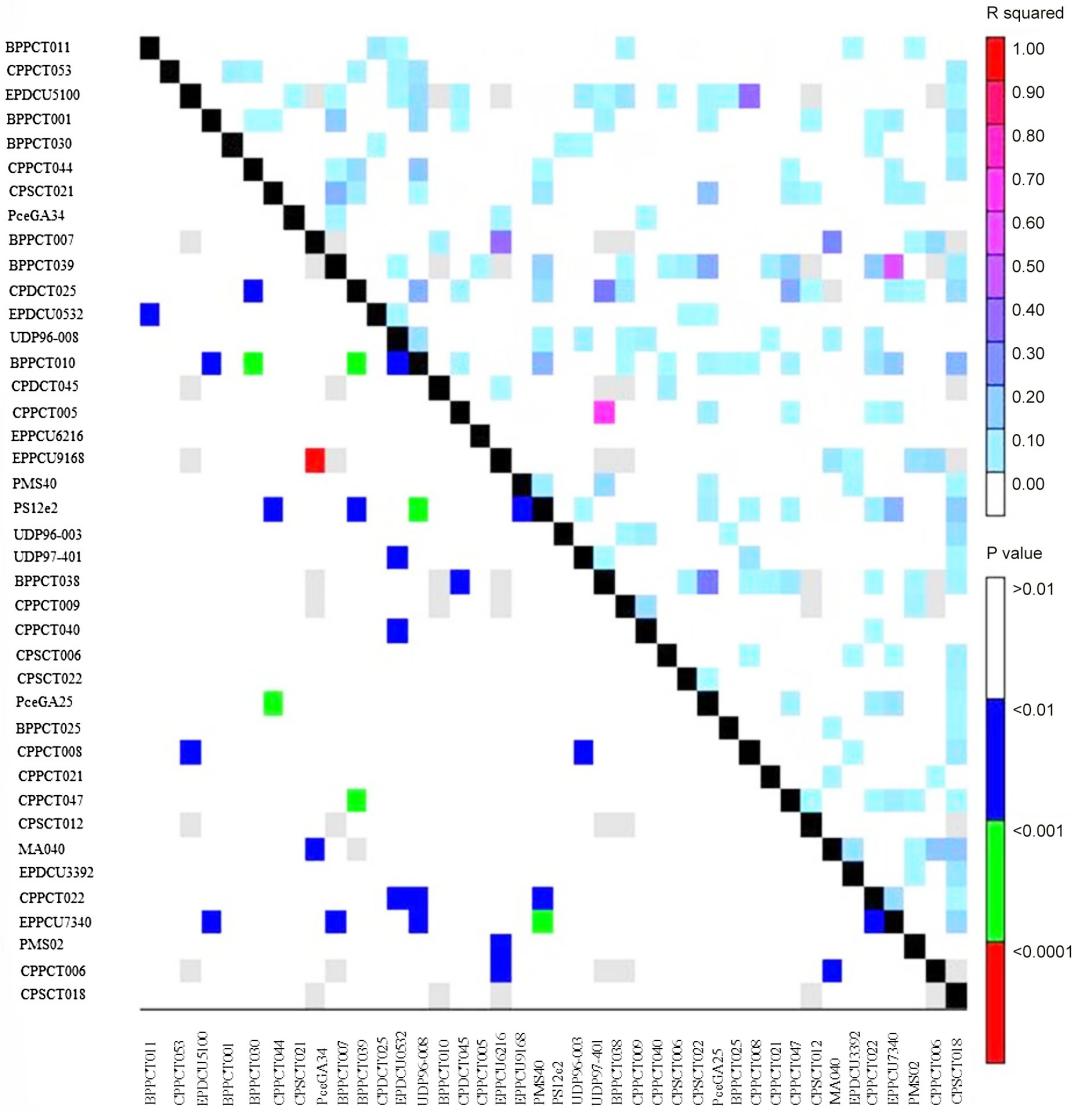
Linkage disequilibrium plot based on 40 SSR markers screened on 98 almond accessions.

Linkage disequilibrium (LD) was estimated after removing low frequency alleles (MAF ≤ 0.05) from all loci in the 98 accessions (Table 3 and Fig 4). Results showed a high level of LD up to 20 cM, which dissipated at farther distances. The range of LD in the region from 0 to 10 cM was 0.061 and 0.058 in the Mediterranean vs non-Mediterranean groups, respectively, increasing to 0.087 and 0.079 in the region from 10 to 20 cM, and finally decreasing to 0.045 and 0.039 in the region between 20 and 30 cM. A high level of LD up to 20 cM was also observed in the Mediterranean and non-Mediterranean accessions. The *r* ^*2*^ values for all intra-chromosomal and inter-chromosomal loci pairs were similar (0.040 vs 0.036). Unlinked marker pairs showed a similar percentage of significant LD in Mediterranean (*r* ^*2*^ values of 0.091 intra-chromosomal and 0.082 for inter-chromosomal loci pairs) and in non-Mediterranean accessions (*r* ^*2*^ values of 0.073 for intra-chromosomal and 0.062 for inter-chromosomal marker loci pairs). The overall level of LD detected was low, which could be mostly likely due to poor marker coverage.

**Table 3:** LD based on r^2^, averaged for map distance classes and germplasm groups based on population structure analysis in the STRUCTURE.

Genomic región (cM)	N*	All accessions (r^2^)	Meditteranean accessions (r^2^)	Non-Mediterranean accessions (r^2^)
0–10	20	0.034	0.061	0.058
10–20	24	0.079	0.087	0.079
20–30	21	0.036	0.045	0.039
>30	23	0.028	0.027	0.032
Intra-chromosomal	88	0.040	0.091	0.073
Inter-chromosomal	692	0.036	0.082	0.062

* Number of marker pairs included in each class. The analysis of linkage disequilibrium (LD) revealed a high level of LD up to 20 cM, with LD decaying after 20 cM.

### Associations between traits and SSR markers

Association analysis was performed for nut and kernel traits in the 98 almond accessions. Marker-trait associations were obtained for chemical traits (Table 4 and S1 Table) and physical traits (Table 5 and S1 Table). We tested five models in TASSEL to determine associations and to also account for the influence of population structure by comparing their ability to reduce the inflation of false positive associations. The *P*-values were plotted in a cumulative fashion for each model and the distribution examined. According to Stich et al. [28] the distribution of *P*-values ideally should follow a uniform distribution with less deviation from the expected *P*-values.

**Table 4:** Statistical significance of the *p* values and associations observed between markers and chemical traits in 98 almond cultivars.

SSR	LG	%var (r^2^) ^(^ ^b^ ^)^	α-T	δ-T	γ -T	Oleic	Linoleic	Stearic	Palmitic	Oil	Protein
BPPCT011	1	70.1	^(^ ^a^ ^)^ **	^(^ ^a^ ^)^ ***	^(^ ^a^ ^)^ **			^(^ ^a^ ^)^ **			
CPDCT025	3	92.9		***	***	***	***	**	***		***
UDP96-003	4	66.7			^(^ ^a^ ^)^ ***	***	**			***	
EPDCU3392	7	66.4									^(^ ^a^ ^)^ **

For multiple testing of genotypes, Bonferroni correction [29] was applied. The *p* values for associations are considered when at least one allele is associated with the SSR.

*p<0.00001

**p = 0.00001–0.0001

***p = 0.0001–0.0012, (K-model)

^(a)^Associations observed in the same regions where QTLs had previously been identified [3, 30]

^(b)^Percentage of the phenotypic variation (r^2^) explained by each marker

**Table 5:** Statistical significance of the *p* values and associations observed between markers and nut and kernel physical traits of almond.

			Nut					Kernel				
SSR	LG	%var (r^2^) ^(^ ^b^ ^)^	W	T	L	Weight	T/L	T	L	Weight	T/L	Size
BPPCT011	1	66.2					^(^ ^a^ ^)^ **		^(^ ^a^ ^)^ *			
EPDCT0532	3	52.2	***	***			***					***
CPSCT006	5	55.4			^(^ ^a^ ^)^ ***		^(^ ^a^ ^)^ **		^(^ ^a^ ^)^ ***			
CPPCT021	6	61.8			^(^ ^a^ ^)^ **			^(^ ^a^ ^)^ **	^(^ ^a^ ^)^ ***			

For multiple testing of genotypes, Bonferroni correction [29] was applied. The *p* values for associations are considered when at least one allele is associated with the SSR. Abbreviations: W, width; T, thickness, L, length

*p<0.00001

**p = 0.00001–0.0001

***p = 0.0001–0.0012, (K-model)

^(a)^Associations observed in the same regions where QTLs had previously been identified [3, 30]

^(b)^ Percentage of the phenotypic variation (r^2^) explained by each marker

The association analysis using the GLM approach (being the naïve model), Q-model and P-model, detected a large number of associations between the markers and phenotypes which after Bonferroni correction for multiple testing of accessions, reduced down the number of associations. The Q-model (GLM with Q-matrix as correction for population structure) showed 50 associations for chemical traits and 32 for physical traits. It appears that these models may not have accounted for the heterogeneity of the genetic background, which may have resulted in false positive associations.

**Fig 5:**
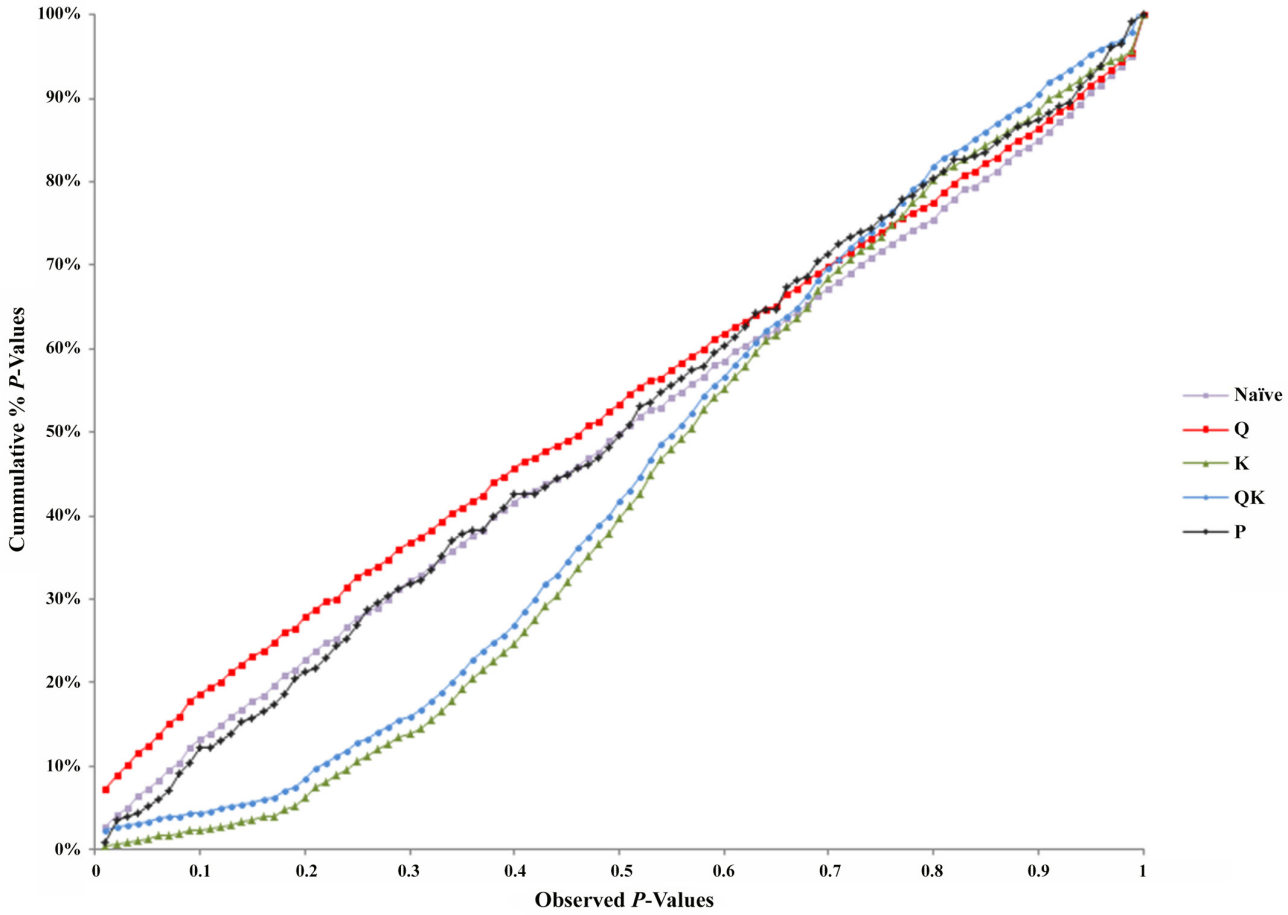
Comparison of different genome wide association study (GWAS) models. Cumulative distribution of *P*-values was computed from the DNA markers and phenotypes for the different association models.

The K-model (MLM with K-matrix as correction for population structure) and QK-model (MLM with Q-matrix and K-matrix as correction for population structure) showed good fit for the *P*-values (*P* < 0.001), while the other models were characterized by the excess of small *P*-values (abundance of spurious associations) (Fig 5). These two later models showed high uniform distribution of *P*-values.

Taking into account the performance of the different models, only results from the K-model will be presented and discussed here since this appeared to have controlled population structure and kinship relationships better. Sixteen significant associations were detected between four SSRs and nine chemical traits (Table 4). The BPPCT011 locus was associated with α-T, δ-T, γ –T and stearic acid, the CPDCT025 with δ-T, γ –T, oleic, linoleic, stearic and palmitic acids and protein content, UDP96-003 with γ –T, oleic and linoleic acids, and oil content, and finally, EPDCT3392 with protein content. For physical traits, 12 marker-trait associations were identifiedsuch as BPPCT11 with nut T/L and kernel lengh, EPDCT0532 with nut weight, thickness and T/L, and kernel size, CPSCT006 with nut length and T/L, and kernel length, and CPPCT021 with nut lenght and thickness, and kernel length. The association analysis performed using the mixed linear model (MLM) showed results consistent with those identified by the GLM approach. The percentage of phenotypic variation explained by these markers ranged between 92.9 and 66.4%, with CPDCT025 having the maximum value and EPDCT3392 the minimum value. The *P*-values showing the level of significance of the associations between SSR markers and phenotypic traits are shown in Fig 5. The percentage of phenotypic variation explained by each marker ranged between 99.5 and 24.6%, with UDP96-003 having the maximum value and PMS2 the minimum value. Please note that some associations were observed in the same regions where QTLs had previously been identified (Tables 4 and 5).

## Discussion

### Genetic diversity

Over the last decade, SSR markers have proven to be a marker of choice in many breeding programs due to their abundance, co-dominance and feasibility. These markers are still being used today for linkage mapping and genetic diversity analysis in many crop plants, such as almond [12, 31]. In the present work, we used a set of 40 genomic SSR markers spanning the almond genome to evaluate population structure and to identify the genetic mechanisms underlying inheritance of chemical and physical almond seed traits. Our plant material consisted of 98 almond accessions from different countries maintained at the CITA almond collection.

A total of 557 alleles were obtained from 40 SSR loci, with a mean of 13.9 alleles. This average allele number, when compared with other almond studies, is lower than 18.7 obtained by Fernandez i Marti et al. [12], but very similar to the 14.6 reported by Elhamzaoui et al. [31], and much higher than 4.7 obtained by Martínez-Gómez [32]. The reason for this discrepancy in the mean number of alleles (18.66 *vs* 13.9) could be because wild genotypes were used in Fernandez i Marti et al. [12], which resulted in more alleles some of which are novel, whereas mainly domesticated germplasm were used in this study. Also, the F values were positive in 33 loci but negative in 7 loci in this study, indicating a high level of heterozygosity as would be expected in a self-incompatible species such as almond. The average heterozygosity of 0.66 observed in this study was very similar to 0.62 obtained by Fernandez i Marti et al. [12], but slightly higher than 0.59 obtained by Elhamzaoui et al. [31] and 0.59 obtained by Martínez-Gómez [32]. These results suggest that our plant material showed enough variability and diversity suitable for association mapping.

### Population structure and LD

The complex breeding history of many important crops and the limited gene flow in most wild plants have created complex stratification within the germplasm [33]. In many fruit species, domestication occurred relatively late, so the bottlenecks may have been relatively recent and of short duration [34]. Hence, despite the diversity observed in almond, genetic bottlenecks may have occurred during almond dissemination.

**Fig 6:**
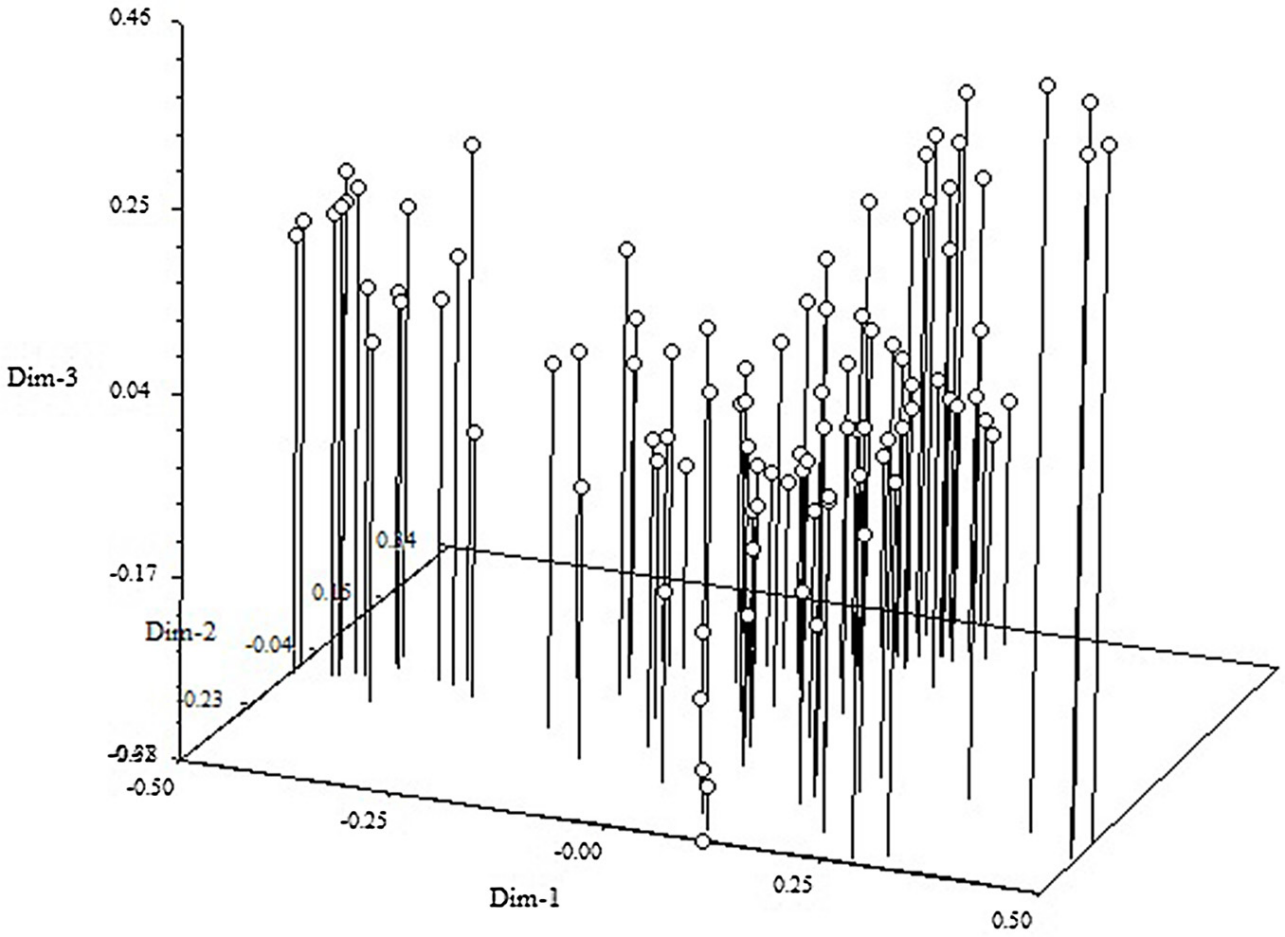
Principal component analysis (PCA) plot showing the grouping of the almond accessions.

The presence of population stratification and unequal distribution of alleles could result in non-functional, spurious associations [35]. In order to understand the distribution of genetic diversity in the almond cultivars, we used a model-based clustering approach as implemented in ‘STRUCTURE” to infer population structure. The results showed two separate pools of cultivars, the Mediterranean and non-Mediterranean, which are in line with those obtained by Fernandez i Marti et al. [12], where only 17 SSRs were used. In addition, the cultivars from Europe grouped separately from the Asian, American and Australian cultivars in Fernandez i Marti et al. [12]. We also performed principal component analysis (PCA) in TASSEL and obtained similar results (Fig 6). The first ten principal components explained a cumulative variation of 64.26%.

Linkage disequilibrium (LD) was estimated after eliminating low frequency alleles (MAF ≤ 0.05) from all loci in the 98 accessions (Table 3). A LD up to 20 cM was observed which dissipated at farther distances. The range of LD in the region from 0 to 10 cM was 0.061 and 0.058 in the Mediterranean vs non-Mediterranean groups, respectively, increasing to 0.087 and 0.079 in the region from 10 to 20 cM, and finally decreasing to 0.045 and 0.039 between 20 and 30 cM. A high level of LD up to 20 cM was also observed in the Mediterranean and non-Mediterranean accessions. The *r* ^*2*^ values for all intra-chromosomal and inter-chromosomal loci pairs were similar (0.040 vs 0.036). Unlinked marker pairs showed a similar percentage of significant LD in the Mediterranean (*r* ^*2*^ values of 0.091 for intra-chromosomal and 0.082 for inter-chromosomal loci pairs) and in the non-Mediterranean groups (*r* ^*2*^ values of 0.073 for intra-chromosomal and 0.062 for inter-chromosomal marker loci pairs). The overall level of LD detected was low, which could probably be attributed to poor marker coverage.

For cultivated species, it is generally observed that domestication and breeding have a strong impact on the level of genetic diversity in populations. In addition, domestication and breeding are expected to influence the level of LD within a population. Not surprisingly, we detected a rapid decline of LD in our almond cultivars. Nevertheless, the decline of LD in almond seems to be relatively comparable to previous studies based on sequence data in self-incompatible crop species. For example, LD decays to negligible levels within 150–750 bp in wild tomatoes [36] and within 0.2–1.5 kbp in maize [37]. In addition, the extent of LD we observed in the self-incompatible almond species is comparable to the extent of LD recently published in other almond studies (*r* ^*2*^ = 0.04 for intra-chromosomal regions and *r* ^*2*^ = 0.03 for inter-chromosomal regions) [12], or in the self-incompatible sweet cherry (*r* ^*2*^ = 0.028) [38], and in self-compatible peach (*r* ^*2*^ = 0.041 for intra-chromosomal regions and *r* ^*2*^ = 0.028 for inter-chromosomal regions) [22]. On the whole, direct comparisons of the extent of LD among studies should be treated with caution due to use of different LD measurement parameters and/or molecular marker systems in different studies.

Although LD in general decays more rapidly in an outcrossing species than in a selfing species, since recombination may be less effective in a selfing species [39], the level of LD decay observed in our study is comparable to the decay found in self-compatible *Prunus* species such as peach [19, 22]. Population structure influences the magnitude and pattern of LD in almond, as in other species, such as *Arabidopsis thaliana* [40], maize [37], and barley [41]. The population structure observed among the different almond groups in this study could be responsible for the level of LD detected. A significant effect of domestication on the LD extent has been demonstrated in several crop plants due to bottlenecks and genetic drift [42]. Use of small sample sizes is expected to bias LD estimations [43]. The small sample size coupled with the low numbers of markers in our study could also explain why we observed similar extents of LD to the self-compatible peach [22].

In the presence of LD, it will be possible to identify genetic regions (if LD extends to a distance of several centi Morgans) or genes (if LD decays quickly, in a few thousand base pairs) associated with a particular trait of interest by genome-wide associations or by individual SNPs (Single Nucleotide Polymorphisms) or SNP haplotypes within a candidate gene [44], respectively.

### Association Analysis

Association mapping is increasingly being utilized to detect marker-QTL linkage associations using plant materials routinely developed in breeding programs. Compared to conventional QTL mapping approaches, association mapping could be a more practical approach for cultivar development, considering that markers linked to major QTLs can immediately be utilized in marker assisted selection (MAS), once new QTLs are identified. In general, association mapping may more suited to organisms with little or no pedigree information, populations with rich allelic diversity, moderate to high nucleotide diversity, and for traits with little or no selection history and also controlled by many loci with small effects, in addition to having older alleles with low frequencies [45, 46, 47]. These situations appear applicable to vegetatively propagated fruit trees, particularly almond, with a high level of heterosis and a short breeding history [1].

Limited information exists in *Prunus* species on QTLs linked to nut and kernel traits. In sweet cherry, [48] identified three QTLs linked to fruit size on LG2 and LG6 using SSR markers, while in peach, [49] identified one QTL linked to fruit weight on LG1 using RFLP, AFLP, RAPD, and SSR markers. [30], in their first complete genetic framework map for physical traits of almond identified for the first time, the association of physical parameters of almond nut and kernel, with 14 putative QTLs. More studies are needed in this area to facilitate QTL co-localization and/or synteny analysis among the *Prunus* species, with a view to undergoing candidate gene identification and fine mapping.

In our study, microsatellite markers were located in regions where QTLs had previously been identified using conventional QTL mapping approaches [3, 30]. This suggests that association mapping using plant materials routinely developed by breeders can effectively detect major QTLs. The SSR loci associated with chemical and physical traits in our association analysis included five DNA markers (BPPCT011, UDP96-003, EPDCT3392, CPSCT006 and CPPCT021) that were previously identified for linkage mapping and QTL identification [3, 30].

Regarding chemical traits, a total of 16 associations were found between four DNA markers and nine chemical traits phenotyped. BPPCT011 located on LG1 was associated with α- tocopherol, δ- tocopherol, γ-tocopherol and stearic acid. The map positions for these marker-trait associations are in agreement with a previous study [3], which reported a QTL mapped close to BPPCT011, and also associated with three tocopherol compounds and stearic acid. Additional associations were found between γ-tocopherol and UDP96-003 (LG4). This association is also in agreement with the QTLs identified for the same traits by Font i Forcada et al. [3]. Similarly, protein content showed association with the locus EPDCT3392 located on LG 7, which was in agreement with the QTL found at the same position as Font i Forcada et al. [3]. In addition, this study identified other loci associated with δ- tocopherol, γ-tocopherol, oleic, linoleic, stearic, palmitic fatty acids and protein content on LG3 (CPDCT025); and oleic, linoleic and oil content on LG4 (UDP96-003) that were not reported by Font i Forcada et al. [3]. The discrepancies in marker-locus-trait associations between the two studies could be attributed to number of marker loci used. In addition, this study used a large outbred population which invariably has more meiotic recombination events and a higher number of alleles segregating unlike [3], who used a bi-parental mapping population where only two alleles are expected to segregate at any locus.

Font i Forcada et al. [3] proposed candidate genes including Acyl-CoA (controlling the synthesis of long-chain saturated fatty acids), Enoyl-CoA hydratase (controlling the two main fatty acids such as oleic and linoleic) or ACP (controlling stearic acid) for the fine mapping of almond quality components. In the present work, the association found between α- tocopherol, δ- tocopherol, γ-tocopherol and stearic acid and the marker BPPCT011 on LG 1 appears to map within the interval of the Acyl-CoA gene (ppa002255m). This gene is very close to CPPCT042, located also on LG1 [3]. Additionally, three more candidate genes located on LG 3, LG 4 and LG 7 have been identified to control protein ligase and fatty acid content in plant cells (ppa002006m, ppb019158 and ppa024762), and appear to be within the interval flanked by the SSRs CPDCT025, UDP96-003 and EPDCT3392, which are associated in our study with tocopherols, fatty acids, oil and protein content. Since this is the first association mapping in almond, we suggest further studies including QTL validation and fine mapping to identify the causative polymorphisms involved in fatty acid variation.

On the other hand, a total of 12 associations related to physical nut and kernel traits were identified in this study. One of the associations was between the marker CPSCT006 and nut length and T/L ratio, and kernel length, which is corroborated by the QTLs identified on LG 5 (CPSCT006) by Fernández i Martí et al. [30]. The other association was between the marker BPPCT011 (LG 1) and the nut T/L ratio and kernel length. This association maps close to the QTL (CPPCT042) for the same trait found on LG1 by Fernández i Martí et al. [30]. Locus CPPCT021 (LG 6), associated with nut length and thickness and kernel length in this study, was also found in Fernández i Martí et al. [30]. This association also agrees with the QTL found in the same position by Fernández i Martí et al. [30]. Other important associations observed in this study were between the marker EPDCT0532 (LG 3) and nut width, thickness and T/L and kernel size, and this is in agreement

Compared to peach and apple, almond has a poorly developed genomic infrastructure. In order to accelerate association studies in this crop, a large-scale SNP discovery may be required. Recent advances in genomic tools, including genome sequencing and high-density SNP genotyping [50], have enabled the development of new approaches for mapping complex traits. The identification of causal genes underlying specific traits is a major goal in plant breeding, subsequently offering opportunities to develop genomic selection tools. Next generation sequencing (NGS) is playing an increasingly significant role in facilitating SNP discovery in less-characterized fruit tree species. Moreover, due to the abundance of SNPs within a genome, and the availability of high-throughput sequencing methods, SNPs are increasingly becoming one of the commonly used markers for genotyping horticultural species.

Apple [51] and peach [52] genomes, two of the most important fruit tree species, have been sequenced in the last few years, thus facilitating the rapid design of high-throughput SNP genotyping assays such as the 1536 SNP GoldenGate for apple [24] and the 9000 SNP Infinium chip for apple and peach [53, 54] and the 6000 SNP Infinium chip for cherry [55]. These chips are supposed to provide excellent opportunities for candidate gene-based association mapping. However, in almond, the application of NGS technologies and bioinformatic tools to generate high frequency single nucleotide polymorphisms still remains unexplored. Hence, the use of high-density genetic linkage maps based on SNP markers in genome mapping and phenotypic selection is still very limited in almond germplasm and breeding populations. Thus expressed sequence tags (EST) and microsatellite (SSR) are the more common sources of molecular markers used for association mapping [24].

It is worthy to note that an international consortium has recently been created in 2014 to sequence the whole genome of the almond cultivar ‘Texas’ for intended release at the end of 2015 (Almond International Consortium). This advancement in genome sequencing will offer important new possibilities for SNP discovery and genome wide association studies (GWAS) in this nut crop. The cost of growing and maintaining tree crops until they reach maturity is very high, thus any effort to carry out early selection will be highly desirable to reduce orchard costs at the seedling stage.

In the few studies on association mapping in other *Prunus* species, such as peach [19, 22] and sweet cherry [23], the authors used mostly SSR markers. Marker numbers ranged from 15 in Ganopoulos et al. [23] to 40 in Font i Forcada et al. [22] and then 53 in Cao et al. [19]. Based on our results we believe that the 40 SSR markers we used may appear adequate for association mapping in almond. Of a total of 28 (16 for chemical and 12 for physical traits) significant associations, more than 10 have been confirmed previously through linkage analysis and QTL mapping. These associations should be followed up using MAS strategies to improve efficiency of selection in breeding programs. The remaining marker-locus-trait associations identified using new markers should be validated in each specific breeding program before deployment for MAS. This study reports for the first time an association study in almond for nut and kernel traits. We suggest that association mapping is a very powerful and complementary approach to conventional mapping approaches for identifying marker-locus-trait associations.

## Conclusions

Our results provide new details about population structure and linkage disequilibrium in a self-pollinating crop like almond. The low level of LD observed in this study is consistent with the broad nature of our germplasm and the breeding history of almond. These results provide an insight into the genetic architecture of important fruit quality traits for almond (tocopherol content, fatty acids, oil and protein content). A total of 28 significant associations between SSR markers and chemical and physical nut and kernel traits were detected in this study. Among them, six chemical and eight physical associations were the same as in previous studies based on conventional QTL mapping. These results will allow for improved efficiency in breeding for quality in almond, and in particular, the simultaneous selection for physical and chemical traits. This study also identified many new marker-locus trait associations, which may be useful for predictions of genetic progress in a breeding program. These associations would provide an efficient platform for classical and MAS breeding in almond.

## Methods

### Plant material and phenotypic evaluation

A germplasm collection of 98 almond (*Prunus amygdalus* Batsch) cultivars encompassing a wide range of geographic origins was used (S2 Table). This set included 56 native local Spanish cultivars and 42 cultivars mostly from the USA, France, Greece, Italy, and Portugal but also from Algeria, Argentina, Australia, Bulgaria, Tunisia and Ukraine. All these genotypes are located and maintained at the public Almond Germplasm Bank of the Centro de Investigación y Tecnología Agroalimentaria de Aragón (CITA) located at Zaragoza (Spain). The trees were grown under continental Mediterranean conditions within the Ebro Valley, NE Spain [13].

Phenotypic evaluation of physical nut and kernel traits were performed over a period of three years based on approximately 50 mature fruits randomly collected from each tree, as described in Font i Forcada et al. [7]. The fruit was considered mature when the mesocarp was fully dry and split along the fruit suture while the peduncle was near to complete abscission [56]. After discarding the mesocarp, the nuts were left at room temperature for 2–3 weeks. Following nut measurements, shells were cracked to expose the kernel, the width (W), thickness (T), length (L), and weight of which were measured with a digital calliper with a precision of 0.01 mm. These variables allowed the estimation of the T/L ratio and the size dimensions (L x W x T) for the kernels.

For chemical traits, the oil was extracted from 3 g of ground almond kernels using a Soxtec Avanti 2055 fat extractor (Foss Tecator, Höganäs, Sweden). Later, butylated hydroxytoluene methanolic solution was added as an antioxidant agent. The oil extraction was duplicated using 30 fruits from each genotype. These analyses were carried out during three years. The oil sample was used first to prepare the methyl esters of the corresponding fatty acids (FAMEs; oleic, linoleic, palmitic, stearic and palmitoleic). The FAMEs were separated using a gas chromatograph HP 6890 and detected using a flame ionization detector, equipped with a capillary column (HP-Innowax 30 m x 0.25 mm i.d.) and 0.25 μm film thickness (Agilent Technologies, Waldbronn, Germany). Tocopherol content (α, δ and γ) was determined by high performance liquid chromatography (HPLC) according to a modification of the method described by López-Ortiz et al. [57]. Finally, the protein content was determined from the total N content obtained by the Dumas method [58] and applying a conversion factor as shown: % Protein = Kc * % total Nitrogen (Kc = 6.25). A sample of 0.2 g of almond flour was weighed and introduced into the analyzer LECO FP-528 Protein/Nitrogen Analyzer (LECO Corporation, Saint Joseph, MI, USA).

### Genotyping

Genomic DNA was isolated using the PowerPlant DNA isolation Kit (MO BIO Laboratories, CA, USA). The DNA was quantified and diluted to 10 ng uL^-1^ for PCR amplifications. For association mapping and genetic diversity analysis, 40 sets of fluorescently labeled SSR primers (PET, NED, VIC and 6-FAM) were subsequently used to genotype the 98 accessions as they showed high polymorphism at a single locus. These primers previously developed in peach, plum, sweet cherry and almond (S1 Table) spans the eight *Prunus* linkage groups. Polymerase chain reactions were performed in a 20 uL volume containing 1× PCR buffer, 1.5 mM MgCl_2_, 0.2 mM dNTPs, 0.2 μM of each primer, one unit of Taq DNA Polymerase (Invitrogen, Madrid, Spain) and 20 ng of genomic DNA. The thermocycling programme included denaturation for 1 min at 94°C, followed by 35 cycles of 15 s at 94°C, 15 s for the annealing temperatures indicated in S1 Table for the different primers used, and 1 min at 72°C, and a final extension of 2 min at 72°C. The PCR reactions were carried out in a 96-well block thermal cycler (Applied Biosystems, Madrid, Spain). Each reaction was repeated and analyzed twice to ensure reproducibility and the size standard used in the sequencer was Gene Scan 500 Liz (Applied Biosystems). Sequencing was done using an AB3130xl DNA analyzer and fragment analysis was done using the GeneMapper software v4.1 (Applied Biosystems). S1 Table shows the details of the SSR primer pairs.

### Genetic diversity analysis

The data obtained with the 40 SSRs allowed the calculation of several genetic parameters including the number of alleles per locus (A), the number of effective alleles [A_e_ = 1/(1-H_e_)], the observed heterozygosity (H_o_ = number of heterozygous individuals/number of individuals scored), the expected heterozygosity (H_e_ = 1-∑ρ_i_^2^, where ρ_i_ is the frequency of the i^th^ allele), and the Wright’s fixation index (F = 1-H_o_/H_e_) for comparing both heterozygosities. All parameters were estimated using the PopGene 1.31 software. Phylogenies were estimated using the Neighbor-joining method [59]. Neighbor joining analyses were conducted with the PAUP* v.4.0b10 phylogenetics package [60] to generate an un-rooted phylogenetic tree (Fig 1).

To determine population structure and/or identify the number of subgroups in the almond germplasm, different methodologies and software packages were employed and compared. For quantitative assessment of the number of groups in the panel, a Bayesian clustering analysis was performed using a model-based approach implemented in the software package STRUCTURE v 2.3 [27]. It was run under the assumption of admixture with independent allele frequencies. Analyses were performed for the number of subgroups ΔK (where K specifies the number of subpopulations or clusters), based on the rate of change in the log probability of the data [26] ranging from one to ten. We performed twenty independent runs per K value starting with 10,000 burn-in period and 100,000 MCMC replications. A burn-in of 20,000 and 250,000 Markov Chain Monte Carlo replications seemed to be the best fit for the data at K = 3. A model-based clustering algorithm was applied to identify subgroups with distinctive allele frequencies. In the second approach, principal component analysis (PCA) based on the dissimilarity matrix was performed using TASSEL.

Low frequency alleles (considering MAF ≤ 0.05) were removed before estimation of linkage disequilibrium (LD) between pairs of multi-allelic loci for the same or different linkage group (LG), using the *r* ^*2*^ coefficient (TASSEL 3.0; [46]). The statistical *r* ^*2*^ gives an indication of both recombination and mutation [15]. The significance level of LD between loci was examined using a permutation test implemented in TASSEL for multiallelic loci, using the “rapid permutation” option.

### Association analysis

The hypothesis for associating each molecular marker with the trait of interest was tested using the software TASSEL. Different statistical models were used to calculate *P*-values, along with accounting for population structure to avoid spurious associations. Five models comprising both general linear models (GLM) and mixed linear models (MLM) were selected to test for marker-trait-associations. Results were compared to determine the best model. The first ten significant PCs explained 64.26% of the cumulative variance of all markers. The following models were tested: i) Naïve model: GLM without any correction for population structure; ii) Q-model: GLM with Q-matrix as correction for population structure; iii) P-model: GLM with PCs as correction for population structure; iv) QK-model: MLM with Q-matrix and K-matrix as correction for population structure and kinship relationships; v) K-model: MLM with K-matrix as correction for kinship relationships structure [27, 28]. The mean value of the markers at *P* < 0.005 was used for determining the significance of marker-trait associations. We focused the association mapping on all LGs on the *Prunus* reference map. Significant markers were identified using the Bonferroni procedure at the p<0.00125 experimental-wide threshold. Alleles with a frequency (MAF) lower than 5% were removed [61] before data analysis. The critical *P*-values for assessing the significance of marker-trait-associations were calculated based on a false discovery rate (FDR), which was found to be highly stringent. Considering the stringency of the model used for accounting for population structure and cryptic variation due to kinship relationships, most of the false positives were inherently controlled. Among the five models, the best model was selected based on the smallest mean square difference (MSD) between the observed and expected *P*-values, since the random marker *P*-values follow a uniform distribution [62]. To detect significant markers, the phenotypic variation (R^2^) was calculated using a simple regression equation implemented in GLM procedure in TASSEL. Association study was determined using 40 SSRs [63–72].

## Supporting Information

S1 Table Trait associations of the 40 SSR markers used for genotyping 98 almond accessions. (DOCX)

S2 Table Name and origin of the almond accession collected and maintained at the Almond Germplasm Bank of the CITA. (DOCX)

## References

[1] Socias i Company R, Alonso JM, Kodad O, Gradziel TM. Almond In Badenes, ML, Byrne, D, ed Fruit Breeding, Handbook of Plant Breeding. 2012; 8: pp 697–728.

[2] Socias i Company R, Kodad O, Alonso JM, Gradziel TM. Almond quality: a breeding perspective. Hortic Revi. 2008; 34:197–238.

[3] Font i Forcada C, Fernández i Martí A, and Socias i Company R. Mapping quantitative trait loci for kernel composition in almond. BMC Genetics. 2012; 13:47 10.1186/1471-2156-13-47 22720975 PMC3432608

[4] Chen D, Bruno J, Easlon E, Lin SJ, Cheng HL, Alt FW et al Tissue-specific regulation of SIRT1 by calorie restriction. Genes Develop. 2008; 22:1753–1757. 10.1101/gad.1650608 18550784 PMC2492662

[5] Gradziel TM, Wang D Susceptibility of California almond cultivars to aflatoxigenic *Aspergillus flavus* . HortScience. 1994; 29:33–35.

[6] Grasselly C. L’amandier: caractères morphologiques et physiologiques des variétés, modalité de leur transmission chez les hybrides de première génération. PhD thesis. Univ Bordeaux, France; 1972.

[7] Font i Forcada C, Kodad O, Juan T, Estopañán G, Socias i Company R Genetic variability and pollen effect on the transmission of the chemical components of the almond kernel. Spanish J Agric Res. 2011; 9:781–789.

[8] Souza V, Byrne DH, Taylor JF. Heritability, genetic and phenotypic correlations, and predict selection response of quantitative traits in peach. II. An analysis of several fruit traits. J Amer Soc Hortic Sci. 1998; 123: 604–611.

[9] Yao Q, Mehlenbacher A. Heritability, variance components and correlation of morphological and phenological traits in hazelnut. Plant Breed. 2000; 119: 369–381.

[10] Fernández i Martí A, Font i Forcada C, Socias i Company R. Genetic analysis for physical nut traits in almond. Tree Genetics & Genomes. 2013; 9:455–465.

[11] Socias i Company R, Felipe AJ. Almond: a diverse germplasm. HortScience. 1992; 27:717–718.

[12] Fernandez i Marti A, Font i Forcada C, Kamali K, Rubio-Cabetas MJ, Wirthensohn M, Socias i Company. Molecular analyses of evolution and population structure in a worldwide almond [*Prunus dulcis* (Mill.) D.A. Webb syn. *P* *amygdalus* Batsch] pool assessed by microsatellite markers. Genet Resour Crop Evol. 2014. 10.1007/s10722-014-0146-x

[13] Espiau MT, Ansón JM, Socias i Company R. The almond germplasm bank of Zaragoza. Acta Horticulturae. 2002; 591:275–278.

[14] Alonso JM, Ansón JM, Espiau MT, Socias i Company R. Determination of endodormancy break in almond flower buds by a correlation model using the average temperature of different day intervals and its application to the estimation of chill and heat requirements and blooming date. J Am Soc or Hortic Sci. 2005; 130:308–318.

[15] Kodad O, Estopañán G, Juan T, Mamouni A, Socias i Company R. Tocopherol concentration in almond oil: genetic variation and environmental effect under warm conditions. J Agric Food Chem. 2011; 59:6137–6141. 10.1021/jf200323c 21524140

[16] Yu JM, Pressoir G, Briggs WH, Bi IV, Yamasaki M, Doebley JF et al A unified mixed-model method for association mapping that accounts for multiple levels of relatedness. Nat Genet. 2006; 38:203–208. 16380716 10.1038/ng1702

[17] Mackay I, Powell W. Methods for linkage disequilibrium mapping in crops. Trends Plant Sci. 2007; 12:57–63. 17224302 10.1016/j.tplants.2006.12.001

[18] Oraguzie NC, Wilcox PL. An overview in association mapping, In Oraguzie NC, Rikkerink EHA, Gardiner SE, De Silva HN, ed Association mapping in plants, pp 1–9. Springer, New York, USA; 2007.

[19] Cao K, Wang L, Zhu G, Fang W, Chen C, Luo J. Genetic diversity, linkage disequilibrium, and association mapping analyses of peach (*Prunus persica*) landraces in China. Tree Genet Genomes. 2012; 8:975–990.

[20] Flint-Garcia SA, Thornsberry JM, Buckler ES Structure of linkage disequilibrium in plants. Ann Rev Plant Biol. 2003; 54:357–374. 14502995 10.1146/annurev.arplant.54.031902.134907

[21] Jorde L. Linkage disequilibrium and the search for complex disease genes. Genome Res. 2000; 10:1435–1444. 11042143 10.1101/gr.144500

[22] Font i Forcada C, Oraguzie N, Igartua E, Moreno MA, Gogorcena Y. Population structure and marker-trait associations for pomological traits in peach and nectarine cultivars. Tree Genet Genomes. 2013; 9:331–349.

[23] Ganopoulos I, Kazantzis K, Chatzicharisis I, Karayiannis I, Tsaftaris A. Genetic diversity structure and fruit trait associations in Greek sweet cherry cultivars using microsatellite based (SSR/ISSR) and morpho-physiological markers. Euphytica. 2011; 181(2): 237–251

[24] Khan MA, Korban SS. Association mapping in forest trees and fruit crops. J Exp Bot. 2012; 63:4045–4060. 10.1093/jxb/ers105 22511806

[25] Brookfield JFY. A simple new method for estimating null allele frequency from heterozygote deficiency. Mol Ecol. 1996; 5:453–455. 8688964 10.1111/j.1365-294x.1996.tb00336.x

[26] Evanno G, Regnaut S, Goudet J. Detecting the number of clusters of individuals using the software STRUCTURE: a simulation study. Mol Ecol. 2005; 14:2611–2620. 15969739 10.1111/j.1365-294X.2005.02553.x

[27] Pritchard JK, Stephens M, Rosenberg NA, Donnelly P. Association mapping in structured populations. Am J Hum Genet. 2000; 67:170–181. 10827107 10.1086/302959 PMC1287075

[28] Stich B, Mohring J, Piepho HP, Heckenberger M, Buckler ES, Melchinger AE. Comparison of mixed-model approaches for association mapping. Genetics. 2008; 178(3):1745–1754. 10.1534/genetics.107.079707 18245847 PMC2278052

[29] Schulze TG, McMahon FJ. Genetic association mapping at the crossroads: which test and why? Overview and practical guidelines. Am J Med Genet. 2002; 114:1–11. 11840498 10.1002/ajmg.10042

[30] Fernández i Martí A, Font i Forcada C, Socias i Company R. Genetic analysis for physical nut traits in almond. Tree Genet Genomes. 2013; 9:455–465.

[31] Elhamzaoui A, Oukabli A, Charafi J, Moumni M. Assessment of genetic diversity of Moroccan cultivated almond (*Prunus dulcis* Mill DA Webb) in its area of extreme diffusion using SSR markers. Ame J Plant Sci. 2012; 3:1294–1303.

[32] Martínez-Gómez P, Arulsekar S, Potter D, Gradziel TM. An extended interspecific gene pool available to peach and almond breeding as characterized using simple sequence repeat (SSR) markers. Euphytica. 2003; 131: 313–322.

[33] Sharbel TF, Haubold B, Olds T. Genetic isolation by distance in Arabidopsis thaliana: biogeography and postglacial colonization of Europe. Mol Ecol. 2000; 9: 2109–2118. 11123622 10.1046/j.1365-294x.2000.01122.x

[34] Haudry A, Cenci A, Ravel C, Bataillon T, Brunel D, Poncet C et al Grinding up wheat: a massive loss of nucleotide diversity since domestication. Mol Biol Evol. 2007; 24:1506–1517. 17443011 10.1093/molbev/msm077

[35] Pritchard JK, Rosenberg NA Use of unlinked genetic markers to detect population stratification in association studies. Am J Hum Genet. 1999; 65:220–228. 10364535 10.1086/302449 PMC1378093

[36] Arunyawat U, Stephan W, Städler T Using Multilocus Sequence Data to Assess Population Structure, Natural Selection, and Linkage Disequilibrium in Wild Tomatoes. Mol Biol Evol. 2007; 24: 2310–2322. 17675653 10.1093/molbev/msm162

[37] Remington DL, Thornsberry JM, Matsuoka Y, Wilson LM, Whitt SR, Doebley J et al Structure of linkage disequilibrium and phenotypic associations in the maize genome. Proc Natl Acad Sci USA. 2001; 98:11479–11484. 11562485 10.1073/pnas.201394398 PMC58755

[38] Arunyawat U, Capdeville G, Decroocq V, Mariette S. Linkage disequilibrium in French wild cherry germplasm and worldwide sweet cherry germplasm. Tree Genet Genomes. 2012; 8:737–755.

[39] Nordborg M, Tabare S. Linkage disequilibrium: what history has to tell us. Trends Genet. 2002; 18:83–90. 11818140 10.1016/s0168-9525(02)02557-x

[40] Ostrowski MF, David J, Santoni S, McKhann H, Reboud X, Le Corre V et al Evidence for a large-scale population structure among accessions of *Arabidopsis thaliana*: possible causes and consequences for the distribution of linkage disequilibrium. Mol Ecol. 2006; 15:1507–1517. 16629807 10.1111/j.1365-294X.2006.02865.x

[41] Comadran J, Thomas WTB, van Eeuwijk FA, Ceccarelli S, Grando S, Stanca AM et al Patterns of genetic diversity and linkage disequilibrium in a highly structured *Hordeum vulgare* association-mapping population for the Mediterranean basin. Theor Appl Genet. 2009; 119:175–187. 10.1007/s00122-009-1027-0 19415228

[42] Caldwell KS, Russell J, Langridge P, Powell W. Extreme population dependent linkage disequilibrium detected in an inbreeding plant species, *Hordeum vulgare* . Genetics. 2006;172: 557–567. 16219791 10.1534/genetics.104.038489 PMC1456183

[43] Teare MD, Dunning AM, Durocher F, Rennart G, Easton DF. Sampling distribution of summary linkage disequilibrium measures. Ann Hum Genet. 2002; 66:223–33. 12174213 10.1017/S0003480002001082

[44] Malysheva-Otto V, Ganal W, Roder S. Analysis of molecular diversity, population structure and linkage disequilibrium in a worldwide survey of cultivated barley germplasm (*Hordeum vulgare* L). BMC Genet. 2006; 7:6 16433922 10.1186/1471-2156-7-6 PMC1408084

[45] Morgante M, Brunner S, Pea G, Fengler K, Zuccolo A, Rafalski A. Gene duplication and exon shuffling by helitron-like transposons generate intraspecies diversity in maize. Nat Genet. 2005; 37:997–1002. 16056225 10.1038/ng1615

[46] Yu JM, Buckler ES. Genetic association mapping and genome organization of maize. Current Opinion Biotechn. 2006; 17:155–160. 16504497 10.1016/j.copbio.2006.02.003

[47] Oraguzie NC, Wilcox PL, Rikkerink EHA, De Silva HN. Linkage disequilibrium, In Oraguzie NC, Rikkerink EHA, Gardiner SE, De Silva HN, ed Association mapping in plants, pp 11–39 Springer, New York, USA; 2007.

[48] Zhang G, Sebolt AM, Sooriyapathirana S, Wang D, Bink M, Olmstead JW et al Fruit size QTL analysis of an F1 population derived from a cross between a domesticated sweet cherry cultivar and a wild forest sweet cherry. Tree Genet Genomes. 2010; 6:25–36.

[49] Abbott G, Rajapakse S, Sosinski B, Lu Z, Sossey-Alaoui K, Gannavarapu M et al Construction of saturated linkage maps of peach crosses segregating for characters controlling fruit quality, tree architecture and pest resistance. Acta Horticulturae. 1998; 465:41–49.

[50] Varshney RK, Nayak SN, May GD, Jackson SA. Next generation sequencing technologies and their implications for crop genetics and breeding. Trends Biotech. 2009; 27:522–530. 10.1016/j.tibtech.2009.05.006 19679362

[51] Velasco R, Zharkikh A, Affourtit J, Cestaro A, Kalyanaraman A, et al The genome of the domesticated apple (*Malus domestica* Borkh). Nat Genet. 2010; 42:833–839. 10.1038/ng.654 20802477

[52] Verde I, Bassil N, Scalabrin S, Gilmore B, Lawley CT, Gasic K et al Development and evaluation of a 9K SNP array for peach by internationally coordinated SNP detection and validation in breeding germplasm. PLoS One. 2012; 7:4. 10.1371/journal.pone.0035668 PMC3334984 22536421

[53] Chagne D, Krieger C, Rassam M, Sullivan M, Fraser J, André C et al QTL and candidate gene mapping for polyphenolic composition in apple fruit. BMC Genomics. 2012; 12:12. 10.1186/1471-2229-12-12 PMC3285079 22269060

[54] Verde I, Abbot AG, Scalabrin S, Jung S, Shu S, Marroni F et al The high quality draft genome of peach (*Prunus persica*) identifies unique patters of genetic diversity, domestication and genome evolution Nat genet. 2013; 45:487–496. 10.1038/ng.2586 23525075

[55] Peace C, Bassil N, Main D, Ficklin S, Rosyara UR, Stegmeir T et al Development and Evaluation of a Genome-Wide 6K SNP Array for Diploid Sweet Cherry and Tetraploid Sour Cherry. PLoS One. 2012; 7:12. 10.1371/journal.pone.0048305 PMC3527432 23284615

[56] Felipe AJ. El almendro: el material vegetal Lérida: Integrum; 2000.

[57] López-Ortiz MC, Prats-Moya S, Sanahuja AB, Maestre-Pérez SE, Grané-Teruel N, Martín-Carratalá ML Comparative study of tocopherol homologue content in four almond oil cultivars during two consecutive years. J Food Comp Anal. 2008; 21:144–151.

[58] Dumas JBA. Procedes de l'Analyse Organique, Ann Chim Phys. 1831; 247:198–213.

[59] Saitou N, Nei M. The neighbor-joining method: a new method for reconstructing phylogenetic trees. Mol Biol Evol. 1987; 4: 406–425. 3447015 10.1093/oxfordjournals.molbev.a040454

[60] Swofford, DL. PAUP*, phylogenetic analysis using parasimony (*and other methods) Sinauer Associates, Sunderland, MA; 2003.

[61] Wilson LM, Whitt SR, Ibañez AM, Rocheford TR, Goodman MM, Buckler ES. Dissection of maize kernel composition and starch production by candidate gene association. Plant Cell. 2004; 16:2719–2733. 15377761 10.1105/tpc.104.025700 PMC520967

[62] Yu J, Pressoir G, Briggs WH, Bi IV, Yamasaki M, Doebley JF et al A unified mixed-model method for association mapping that accounts for multiple levels of relatedness. Nature Genetics. 2005; 38: 203–208. 16380716 10.1038/ng1702

[63] Dirlewanger E, Cosson P, Travaud M, Aranzana MJ, Poizat C, Zanetto A et al Development of microsatellite markers in peach [*Prunus persica* (L) Batsch] and their use in genetic diversity analysis in peach and sweet cherry (*Prunus avium* L). Theor Appl Genetics. 2002; 105:127–138. 12582570 10.1007/s00122-002-0867-7

[64] Aranzana MJ, Garcia-Mas J, Carbó J, Arús P. Development and variability analysis of microsatellites markers in peach. Plant Breed. 2002; 121:87–92.

[65] Howad W, Yamamoto T, Dirlewanger E, Testolin R, Cosson P, Cipriani G et al Mapping with a few plants: Using selective mapping for microsatellite saturation of the *Prunus* reference map. Genetics. 2005; 171:1305–1309. 16118196 10.1534/genetics.105.043661 PMC1456826

[66] Mnejja M, Garcia-Mas J, Howad W, Badenes ML, Arús P. Simple sequence repeat (SSR) markers of Japanese plum (*Prunus salicina* Lindl) are highly polymorphic and transferable to peach and almond. Mol Ecol Notes. 2004; 4:163–166.

[67] Downey LD, Iezzoni AF. Polymorphic DNA markers in cherry are identified using sequences from sweet cherry, peach and sour cherry. J Am Soc Hortic Sci. 2000; 125:76–80.

[68] Mnejja M, Garcia-Mas J, Howad W, Arús P. Development and transportability across *Prunus* species of 42 polymorphic almond microsatellites. Mol Ecol Notes. 2005; 5:531–535.

[69] Cipriani G, Lot G, Huang HG, Marrazzo MT, Peterlunger E, Testolin R. AC/GT and AG/CT microsatellite repeats in peach [*Prunus persica* (L) Batsch]: isolation, characterization and crossspecies amplification in *Prunus* . Theor Appl Genetics. 1999; 99:65–72.

[70] Cantini C, Iezzoni AF, Lamboy WF, Boritzki M, Struss D. DNA fingerprinting of tetraploid cherry germplasm using SSR. J Am Soc Hortic Sci. 2001; 126:205–209.

[71] Joobeur T, Periam N, de Vicente MC, King GJ, Arús P. Development of a second generation linkage map for almond using RAPD and SSR markers. Genome. 2000; 43:649–655. 10984177

[72] Yamamoto T, Mochida K, Imai T, Shi YZ, Ogiwara I, Hayashi T. Microsatellite markers in peach [*Prunus persica* (L) Batsch] derived from an enriched genomic and cDNA libraries. Mol Ecol Note. 2002; 14:298–301.

