# Supplementary material for: Identification of Genetic Loci Associated with Quality Traits in Almond via Association Mapping
Source: PLoS One. 2015 Jun 25;10(6):e0127656. doi: 10.1371/journal.pone.0127656 (PMC4482440; doi:10.1371/journal.pone.0127656)
Supplement: S1 Table — (DOCX) [file pone.0127656.s001.docx]

**Table S1 Characteristics and trait associations (chemical and physical) of the 40 SSR markers used for genotyping 98 almond accessions. Map distances are based on the T (Texas) (almond) X E (EarlyGold) (peach) reference map.**

| **SSR** | **cM from the top** | **AT** |  |  |  |
| --- | --- | --- | --- | --- | --- |
|  |  |  | **References** | **Chem. trait assoc.** | **Phys. trait assoc.** |
|  |  |  |  |  |  |
|  | **LG 1** |  |  |  |  |
| BPPCT011 | 58.8 | 57 | [63] | α-T, δ-T, γ -T, Stearic | T/L (N), L (K) |
| CPPCT053 | 44 | 58 | [64] | -- | -- |
| EPDCU5100 | 14.5 | 57 | [65] | -- | -- |
|  | **LG 2** |  |  |  |  |
| BPPCT001 | 20.9 | 57 | [63] | -- | -- |
| BPPCT030 | 38 | 57 | [63] | -- | -- |
| CPPCT044 | 7.2 | 58 | [64] | -- | -- |
| CPSCT021 | 39.4 | 46 | [66] | -- | -- |
| PceGA34 | 43.9 | 51 | [67] | -- | -- |
|  | **LG 3** |  |  |  |  |
| BPPCT007 | 11.2 | 57 | [63] | -- | -- |
| BPPCT039 | 18 | 57 | [63] | -- | -- |
| CPDCT025 | 36.4 | 62 | [68] | δ-T, γ -T, Oleic, Linoleic, Stearic, Palmitic, Protein | -- |
| EPPCU0532 | 37.1 | 61 | [65] | -- | W (N), T (N), T/L (N), Size (K) |
| UDP96-008 | 36.4 | 57 | [69] | -- |  |
|  | **LG 4** |  |  |  |  |
| BPPCT010 | 2.1 | 57 | [63] | -- | -- |
| CPDCT045 | 16.8 | 62 | [68] | -- | -- |
| CPPCT005 | 10.4 | 51 | [64] | -- | -- |
| EPPCU6216 | 34.1 | 61 | [65] | -- | -- |
| EPPCU9168 | 2 | 60 | [65] | -- | -- |
| PMS40 | 20 | 55 | [70] | -- | -- |
| PS12e2 | 63 | 55 | [71] | -- | -- |
| UDP96-003 | 28.3 | 57 | [69] | γ -T, Oleic, Linoleic, oil | -- |
|  | **LG 5** |  |  |  |  |
| BPPCT038 | 32.9 | 57 | [63] | -- | -- |
| CPPCT009 | 20.4 | 59 | [64] | -- | -- |
| CPPCT040 | 1.5 | 55 | [64] | -- | -- |
| CPSCT006 | 21.7 | 61 | [66] | -- | L (N), T/L (N), L (K) |
| CPSCT022 | 40.7 | 61 | [66] | -- | -- |
| PceGA25 | 28.4 | 58 | [70] | -- | -- |
| UDP97-401 | 11 | 57 | [69] | -- | -- |
|  | **LG 6** |  |  |  |  |
| BPPCT025 | 56.4 | 57 | [63] | -- | -- |
| CPPCT008 | 8.7 | 58 | [64] | -- | -- |
| CPPCT021 | 83.7 | 61 | [64] | -- | L (N), T (K), L (K) |
| CPPCT047 | 58.9 | 55 | [64] | -- | -- |
| CPSCT012 | 36.2 | 62 | [66] | -- | -- |
| MA040 | 38 | 55 | [72] | -- | -- |
|  | **LG 7** |  |  |  |  |
| CPPCT022 | 18.6 | 50 | [64] | -- | -- |
| EPDCU3392 | 64.7 | 57 | [65] | Protein | -- |
| EPPCU7340 | 0 | 61 | [65] | -- | -- |
| PMS2 | 47.8 | 55 | [70] | -- | -- |
|  | **LG 8** |  |  |  |  |
| CPPCT006 | 24.8 | 59 | [64] | -- | -- |
| CPSCT018 | 1 | 52 | [66] | -- | -- |

Abbreviations used: LG (linkage group), AT (annealing temperature), N (nut), K (kernel), -- (no associations found).
