# Supplementary material for: Identification of Genetic Loci Associated with Quality Traits in Almond via Association Mapping
Source: PLoS One. 2015 Jun 25;10(6):e0127656. doi: 10.1371/journal.pone.0127656 (PMC4482440; doi:10.1371/journal.pone.0127656)
Supplement: S2 Table — (DOCX) [file pone.0127656.s002.docx]

**Table S2 Almond accessions studied, and the country and region of origin.**

| Cultivar | Country of origin | Region | Accession name |
| --- | --- | --- | --- |
| Abizanda | Spain | Huesca | 526 |
| Aï | France |  | 89 |
| Alcina | Spain | Majorca, Balearic Islands | 238 |
| A-S-1 | Spain | Huesca | 80 |
| Aspe | Spain | Zaragoza (CITA) | 518 |
| Aspirilla | Spain | Cuenca | 547 |
| Atocha | Spain | Murcia | 288 |
| Aylés | Spain | Zaragoza (CITA) | 395 |
| Belle d’Aurons | France |  | 339 |
| Belona | Spain | Zaragoza (CITA) | 502 |
| Bertina | Spain | Zaragoza | 448 |
| Biota | Spain | Tarragona | 530 |
| Blanquerna | Spain | Zaragoza (CITA) | 434 |
| Bulbuente | Spain | Zaragoza | 549 |
| Cambra | Spain | Zaragoza (CITA) | 398 |
| Carreirinha | Portugal |  | 319 |
| Cartayera | Spain | Huelva | 383 |
| Castilla | Spain | Huesca | 52 |
| Cavaliera | Italy |  | 20 |
| Chellastone | Australia | South Australia | 260 |
| Colorada | Spain | Murcia | 362 |
| Constantini | Algeria |  | 176 |
| Coop. Mañán | Spain | Alicante | 550 |
| Cosa Nova | Portugal |  | 320 |
| Del Cid | Spain | Alicante-Murcia | 361 |
| Desmayo Largueta | Spain | Huesca-Lleida | 366 |
| Desmayo Rojo | Spain | Huesca-Zaragoza | 364 |
| Dura de Tijarafe | Spain | Palma, Canary Islands | 369 |
| El Paso-4 | Spain | Palma, Canary Islands | 375 |
| Elvira | Spain | Albacete | 193 |
| Emilito | Argentina |  | 511 |
| Exinograd | Bulgaria |  | 387 |
| Ferraduel | France | INRA | 232 |
| Ferragnès | France | INRA | 179 |
| Filippo Ceo | Italy |  | 360 |
| Forastero | Spain | Huelva | 486 |
| Garfi | Spain | Zaragoza (CITA) | 484 |
| Garondès | Spain | Majorca, Balearic Islands | 235 |
| Garrigues | Spain | Murcia | 269 |
| Genco | Italy |  | 257 |
| Glorieta | Spain | IRTA | 494 |
| Guara | Spain | Zaragoza (CITA) | 367 |
| I.X.L. | United States | California | 338 |
| Languedoc | France |  | 341 |
| Lauranne | France | INRA | 473 |
| M. Arbeca | Spain | Lleida | 524 |
| Malagueña | Spain | Málaga | 337 |
| Marcona | Spain | Alicante | 365 |
| Marcona Argentina | Argentina |  | 477 |
| Marconeta | Spain | Huesca | 43 |
| Mardía | Spain | Zaragoza (CITA) | 543 |
| Marta | Spain | CEBAS | 523 |
| Masbovera | Spain | IRTA | 491 |
| Menut | Spain | Majorca, Balearic Islands | 247 |
| Molar de Fuzeta | Portugal |  | 317 |
| Mono | United States |  | 223 |
| Muel | Spain | Zaragoza | 528 |
| Padre Santo | Spain | Palma, Canary Islands | 377 |
| Pané-Barquets | Spain | Lleida | 217 |
| Pau | Spain | Majorca, Balearic Islands | 234 |
| Peerless | United States |  | 128 |
| Pestañeta | Spain | Alicante | 267 |
| Phyllis | Greece |  | 355 |
| Picantilli | Greece |  | 166 |
| Primorskij | Ukraine |  | 168 |
| Rachelle | Italy |  | 258 |
| Rameira | Portugal |  | 307 |
| Ramillete | Spain | Murcia | 287 |
| Raposa | Portugal |  | 315 |
| Redonda de Palma | Spain | Palma, Canary Islands | 371 |
| Rof | Spain | Tarragona | 169 |
| Rumbeta | Spain | Alicante | 423 |
| Soleta | Spain | Zaragoza (CITA) | 503 |
| Sovietskij | Ukraine |  | 344 |
| Supernova | Italy | ISF | 497 |
| Symmetrikji | Greece |  | 353 |
| Taiatona | Spain | Majorca, Balearic Islands | 242 |
| Tardive de la Verdière | France |  | 160 |
| Tardy Nonpareil | United States | California | 524 |
| Tarragonès | Spain | IRTA | 493 |
| Tejeda 1 | Spain | Palma, Canary Islands | 376 |
| Tendra amarga | Spain | Alicante | 270 |
| Texas | United States | Texas | 508 |
| Thompson | United States | California | 340 |
| Tioga | United States | California | 226 |
| Titan | United States | California | 251 |
| Tokyo | United States | California | 222 |
| Torreta | Spain | Majorca, Balearic Islands | 510 |
| Truito | Greece |  | 385 |
| Tsotouliu | Greece |  | 335 |
| Tuono | Italy |  | 124 |
| Verdereta | Spain | Majorca, Balearic Islands | 239 |
| Vinagrilla | Spain | Majorca, Balearic Islands | 245 |
| Vivot | Spain | Majorca, Balearic Islands | 241 |
| Yaltinskij | Ukraine |  | 170 |
| Yosemite | United States | California | 225 |
| Zahaf | Tunisia |  | 324 |
| Zinia | Spain | Zaragoza | 295 |
